# Access to food markets, household wealth and child nutrition in rural Cambodia: Findings from nationally representative data

**DOI:** 10.1371/journal.pone.0292618

**Published:** 2023-10-18

**Authors:** Cam Duong, Shivani Patel, Hung Nguyen-Viet, Rortana Chea, Sinh Dang, Sothyra Tum, Usha Ramakrishnan, Melissa F. Young

**Affiliations:** 1 Doctoral Program in Nutrition and Health Sciences Program, Laney Graduate School, Emory University, Atlanta, Georgia, United States of America; 2 Hubert Department of Global Health, Emory University, Atlanta, Georgia, United States of America; 3 Animal and Human Health Program, International Livestock Research Institute, Nairobi, Kenya; 4 National Animal Health and Production Research Institute, General Directorate of Animal Health and Production, Phnom Penh, Cambodia; 5 Animal and Human Health Program, International Livestock Research Institute, Hanoi, Vietnam; Federal University of Agriculture Abeokuta, NIGERIA

## Abstract

Access to informal fresh food markets plays a vital role in household food security and dietary quality in transitioning rural economies. However, it is not well understood if market access also improves child nutrition and if the improvement applies to all socioeconomic groups. In this secondary research study, we combined a national listing of food markets (n = 503) with a national household survey to examine the associations of market access with diet and height across wealth groups in children aged 6 to 23 months in rural Cambodia. All children under two years of age with dietary data (n = 1537) or anthropometry data (n = 989) were selected from the household survey. Food markets were geocoded using Google Maps or villages’ geographical coordinates publicly available in the Open Development Mekong data platform. Regression calibration was then used to estimate household distance to the nearest market. Descriptive results indicated a highly uneven distribution of food markets with median household distance to the nearest markets ranging between 4 km (IQR: 3–8 km) in the lowland areas and 9 km (IQR: 4–17 km) in the highland areas. Results from the multivariate linear regressions showed that distance to the nearest market was modestly associated with child dietary diversity score (β: -0.17; 95% CI: -0.29, -0.05) but it was not related to child height-for-age z-score, and that household wealth did not modify the associations between distance to markets and child dietary diversity score. These findings suggest that improving access to food markets alone might not lead to meaningful improvement in child diet. Detailed surveys on household food acquisition are needed to clarify the role of food markets relative to other food sources such as subsistence fisheries, subsistence gardening and mobile food traders.

## Introduction

Over the past 10 years since the 2013 Lancet Series on Maternal and Child Undernutrition, global policymakers have galvanized efforts to scale up evidence-based interventions for optimal child nutrition [1, 2]. Despite these efforts, child undernutrition remains a major challenge [3]. In low-and-middle-income countries (LMIC), over 60% of the children under two years of age are given diets lacking nutritious food groups such as eggs, meat, fruits and vegetables [4] and over 20% experience stunted growth [5]. These troubling statistics reflect the need for new approaches to improve child dietary quality and linear growth. While prior approaches have focused primarily on individual behavioral changes [6, 7], nutrition-sensitive agriculture [8] and social protection schemes such as cash transfer or food subsidies [9], global policymakers have recommended strengthening food environment as a novel strategy to improve food access and child nutrition [10, 11].

Informal food markets are a key aspect of food environment in rural LMIC, where households are shifting from growing food crops to cash crops and from farm to non-farm activities [12]. Even among subsistence farmers, such as those in Ethiopia and Malawi, over half of the food consumed at home were acquired from markets [13]. Better access to markets has been theorized to improve household food access through reducing time and cost of food acquisition and increasing household purchasing power via opportunities to acquire modern agricultural inputs at the markets [14]. This hypothesis is supported by several observational studies, which showed better household food access and dietary diversity among households located closer to food markets or food markets centers [15, 16]. A recent intervention study in Ghana evaluated the impact of community development initiatives and showed that enhancing access to food markets significantly increases household food security and nutrition [17]. These findings suggested that better road infrastructure and market development can lead to improvement in household dietary quality.

However, whether improving market access has a meaningful benefit on child nutrition remains uncertain. Out of 28 studies in a systematic review of market access and nutrition [14], only six examined child dietary diversity and anthropometry measures and all of them were carried out in remote farming communities in Ethiopia. Of the six studies, four showed a positive association between better access to market and child dietary diversity [18–21] while the other two noted a null relationship between market access and child height [18, 22]. The null association between market access and child height could be due to the high prevalence of stunted growth in rural Ethiopia such that there is little variation in the height measures to observe the effect of market access. Thus, studies with a wider geographical and socioeconomic heterogeneities are warranted to better examine the role of market access.

In addition, hypothesized benefits of food markets on child nutrition might be conditional on household wealth and purchasing power. Nutritious diets cost much more than diets that meets only energy requirements [23] and also fluctuate in price between 30% and 50% by season [24]. It is thus possible that better access to food markets might benefit households with good purchasing power but offer little advantages to households with low purchasing power. In a study in India, the associations between food market utilization, as measured by household visits to markets, and household food consumption were observed only in the highest wealth quintile [25]. Whether similar observations can be made for child nutrition indicators need to be assessed.

Cambodia offers a unique opportunity to assess the role of food markets on child nutrition indicators. In contrast to under-resourced settings where previous studies of food market access were carried out, Cambodia has experienced a substantial investment in agriculture and rural infrastructure, which allows farmers to diversify their agricultural production and engage in diverse income-generating activities [26]. Such rapid rural development has contributed to significant economic growth, as reflected in the increase in the GDP per capita from US$300 in 2000 and US$1,200 in 2015 [27]. Child undernutrition has also improved accordingly, but it remains a major public health challenge with 22% of the children under the age of five being stunted [28] and 50% not meeting the minimum dietary diversity score [29].

In Cambodia, informal food markets is the primary source of fresh food such as fresh meat, fruits and vegetables, supplying 90% and 50% of the fresh food consumed by urban and rural households, respectively [30]. Food markets are organized into four tiers, which include provincial, district, commune and village food markets. Provincial, district and commune food markets often carry a range of fresh fruits, vegetables, meat and seafood as well as snacks and non-food items, but village food markets vary in quality with some selling limited varieties of vegetables, cooking oils, and snacks and some selling fresh meat and seafood [30]. Besides food markets, rural households also depend on their own home gardens for fresh vegetables and poultry, inland water bodies for fish and seafood, and mobile food traders for fresh meat and vegetables. Despite the presence of these alternative sources, high cost of fresh food and long distance to food markets that carry diverse fresh produce have been documented as serious concerns in the rural areas [30].

In this present study, we use nationally representative data from Cambodia to characterize the distribution of food markets and examine the relationship between market access, household wealth and nutrition in children aged 6 to 23 months in the rural areas. We specifically test two hypotheses: first, better access to market is associated with better child dietary diversity score and height-for-age z-score; and second, the magnitude of these associations is stronger among wealthier households. The results of this study will provide insight on nutrition-sensitive agricultural and rural development policies in the country.

## Materials and methods

### Data sources

In this secondary research study, we obtained cross-sectional child and household data from the Cambodia Demographic and Health Survey 2014 (CDHS) [29] and food market data from Cambodia National Animal Health and Production Research Institute (NAHPRI). In addition, geographical coordinates of Cambodian villages were obtained from Open Development Mekong, a publicly accessible platform that aggregates and publishes data on economic and social development in several Southeast Asian countries including Cambodia [31].

#### Household survey

CDHS is a geocoded, nationally representative household survey that uses two-stage cluster sampling procedure. In the first stage, a sample of clusters were selected with probability proportional to size within each of the 38 strata, which comprises urban and rural strata of 19 provinces or groups of provinces. In the second stage, a fixed number of 24 households were selected from each urban cluster and 28 households from the rural cluster with equal-probability systematic sampling within each cluster. Diet data was collected for the youngest, alive children aged 6 to 23 months, born to women selected for detailed interviews (i.e., de-factor women). Height data was collected for all the children under five years of age, whether or not children were born to de-facto women. We obtained data on child height, child dietary diversity and other sociodemographic information from the child recode file, and data on household ownership of land and livestock from the household recode file.

All households in the same cluster were assigned the same geographical coordinates which were measured at the center of the cluster. Survey clusters’ coordinates were collected using Global Positioning System receivers and were accurate to less than 15 m. Random displacement of survey clusters was introduced to ensure confidentiality with urban clusters displaced within 0–2 km and rural clusters within 0–5 km, except for 1% of rural clusters displaced up to 10 km. Displacement was restricted to ensure that each survey cluster remained within its true district.

#### Food market data

A cross-sectional census of meat-selling food markets was compiled by NAHPRI in 2019 using the data provided by provincial departments of animal health and production, which routinely monitor and manage the safety of food sold in markets. The data included market names, administrative locations (village, commune, district and province) and the number of meat vendors of all 503 informal markets that sell meat such as poultry, pork or beef. As explained earlier, except for some village food markets that sell only vegetables, cooking oils and snacks, other food markets across the village, commune, district and province tiers commonly carry fresh meat, fruits and vegetables. Furthermore, the major food access concern in the rural area is not the distance to any kind of food markets, but rather the distance to quality food markets that have fresh meat available [30]. Thus, by using the list of meat-selling markets, we are unlikely to miss any major fresh food markets. Rather, we gain an advantage of capturing only quality fresh food markets that sell fresh meat and other fresh food, which are matter to household and child nutrition, and do not include food markets that sell primarily dried food items like snacks and cooking oils.

### Data processing

#### Household sample selection

Our initial samples included all children aged 6 to 23 months living in rural area that had information on either dietary diversity score (n = 1594) or height-for-age z-score (n = 1026). We then randomly selected one child from each household with multiple children to obtain samples that had one child per household. Subsequently, we dropped children with missing data on the covariates. The analytical samples included 1537 children with diet data and 989 children with height data.

#### Food market geocoding

Food markets were geocoded in two ways. First, markets’ names and administrative locations were manually searched in Google Maps to retrieve physical addresses. Google Street View was used to verify that a food market existed at the retrieved physical address. Physical addresses were then converted to geographical coordinates using Google Maps’ Place API [32]. Second, if markets were not found in Google Maps, we looked up markets’ administrative locations in the Open Development Mekong’s village dataset [31] and substituted markets’ coordinates by the coordinates of the village where the market was located. Due to the variation in Latin spellings of administrative divisions in Cambodia (for example, Poi Pet vs. Poipet), we retrieved the most matching village by calculating Levenshtein distance [33], a common algorithm used to estimate string similarity. The results were reviewed to verify that the administrative locations were correctly matched. Of 503 markets, 485 markets had coordinates retrieved using Google Maps (n = 95) or substituted by villages’ coordinates (n = 390).

Measures of market density included the count of markets, the count per 10,000 persons and the count per 1000 km^2^ for every region and province. Region- and province-specific population data for density measures was obtained from the Population Census of Cambodia 2019 [34] while area data were calculated using Open Development Mekong’s Cambodia administrative boundary map [31]. These measures of market density were used only for describing food market distribution and not statistical modeling.

Market proximity was the straight-line distance from each household to its nearest food market. The distance was estimated using the regression calibration approach [35] to reduce the impact of CDHS’s displacement procedure, which might lead to the mis-identification of nearest markets and the introduction of random error to the distance calculation. In essence, the regression calibration approach involves displacing the coordinates of household survey clusters multiple times using CDHS’s displacement procedure to generate a validation dataset. The validation dataset was then used to estimate the variance of the random error and subsequently the expected value of the true distance between households and nearest markets. Market proximity was then used for both descriptive analysis and statistical modeling.

### Measurement of key variables

#### Exposures

The primary exposure was market proximity, which was measured as the straight-line distance from households to their nearest food markets. Distance to markets (km) was specified in the natural log scale to improve model fitting in the presence of outliers and influential points.

#### Outcomes

Two primary outcomes included height-for-age z-score and dietary diversity score. We obtained height-for-age z-score directly from CDHS [29], which measured child length/height using standard methods [36] with the use of collapsible length boards that was precise to 1 mm and converted height into height-for-age z-score according to the 2006 WHO child growth standard [37]. The dietary diversity score was derived from the dietary diversity questionnaire, which consisted of Yes/No questions that asked mothers to recall which of the 11 nutrient-rich food items their child consumed in the last 24 hours. The 11 food items were then aggregated to 7 food groups to derive the dietary diversity score that ranged from 0 to 7 [38]. In addition to the primary outcomes, we also assessed the consumption of each of the 11 food items as the secondary outcomes to evaluate which specific food items were related to distance to markets.

#### Covariates

We included socioeconomic characteristics and agricultural ownership measures in our analyses. The socioeconomic characteristics included child age and sex, maternal age (15–24, 25–30, > 30 years), maternal education (no education, incomplete or completed primary education, some secondary education or more), household size (< = 5, > 5 members), household wealth tertile (low, middle, high) and country region (Tonle Sap Lake, Plain, Coastal, and Plateau regions). Maternal age and maternal education were categorized to capture non-linearity of their relationships with child nutrition indicators [39, 40] while household size and household wealth were categorized to minimize the influence of outliers and influential points.

Agricultural ownership measures included the size of agricultural land and the number of livestocks owned by the households. We dichotomized agricultural land size at 1 hectare to obtain a proxy of food security on the basis that most of the sampled households owned none or little agricultural land and, in Cambodia, an hectare of land has been shown as a threshold to meet the milled rice requirement of a family of five [41]. We also calculated Tropical Livestock Unit (TLU) scores, which was developed by the Food and Agricultural Organization to account for the body weight differences between livestock animals and commonly considered a proxy of total value of livestock holdings [42]. The TLU weighting factors were 0.01 for chicken and ducks, 0.1 for goats and sheep, 0.2 for pigs and 0.7 for cows. TLU was then categorized into four categories to represent major typologies of livestock ownership reported in earlier literature, including “no animal”, “mostly small livestock” (e.g. up to 10 chickens)”, “a mixed of large and small livestock” (e.g. 2 pigs, 2 goats, 7 chickens)”, and “owning many animals, mixed of large and small livestock” (e.g. 2 cattle, 2 sheep, 20 chicken or more) [43].

### Statistical strategies

We first described the characteristics of the children included in the analysis using mean and standard deviation for continuous variables and percentages for categorical variables. We then used univariate and multivariate regression models to assess the associations between distance to market and child nutrition indicators. The exposure (distance to markets in natural log scale) and primary outcomes (dietary diversity score and height-for-age z-score) were specified in continuous scale while the secondary outcomes (consumption of individual food items) were specified in binary scale. Linear models were used to model the associations between exposures and primary outcomes. Similarly, we used linear probability models to assess the secondary outcomes instead of the conventional logistic regression approach on the basis that food consumption in our study varied within 20% to 80%, a range where linear probability model yields similar estimates to other non-linear models [44].

In the multivariate models, we decided a priori to adjust for sex, child age, maternal age, maternal education, household size, household wealth, ownership of agricultural land, ownership of livestock and country region. Multicollinearity was assessed in all models. Variance inflation factors > 2 was considered an indication of high collinearity [9].

We further examined the evidence of effect modification by household wealth by introducing an interaction term to the multivariate models of dietary diversity score and height-for-age z-score. We calculated the slope of distance to markets on dietary diversity for each level of household wealth using post-hoc estimations and obtained the overall P-value of the interaction term from the Type 3 test.

All analyses were conducted in R statistical software version 4.1.2 and accounted for the complex survey design [45], and P-values < 0.05 were considered statistically significant.

## Results

### Food market distribution in Cambodia

**Fig 1:**
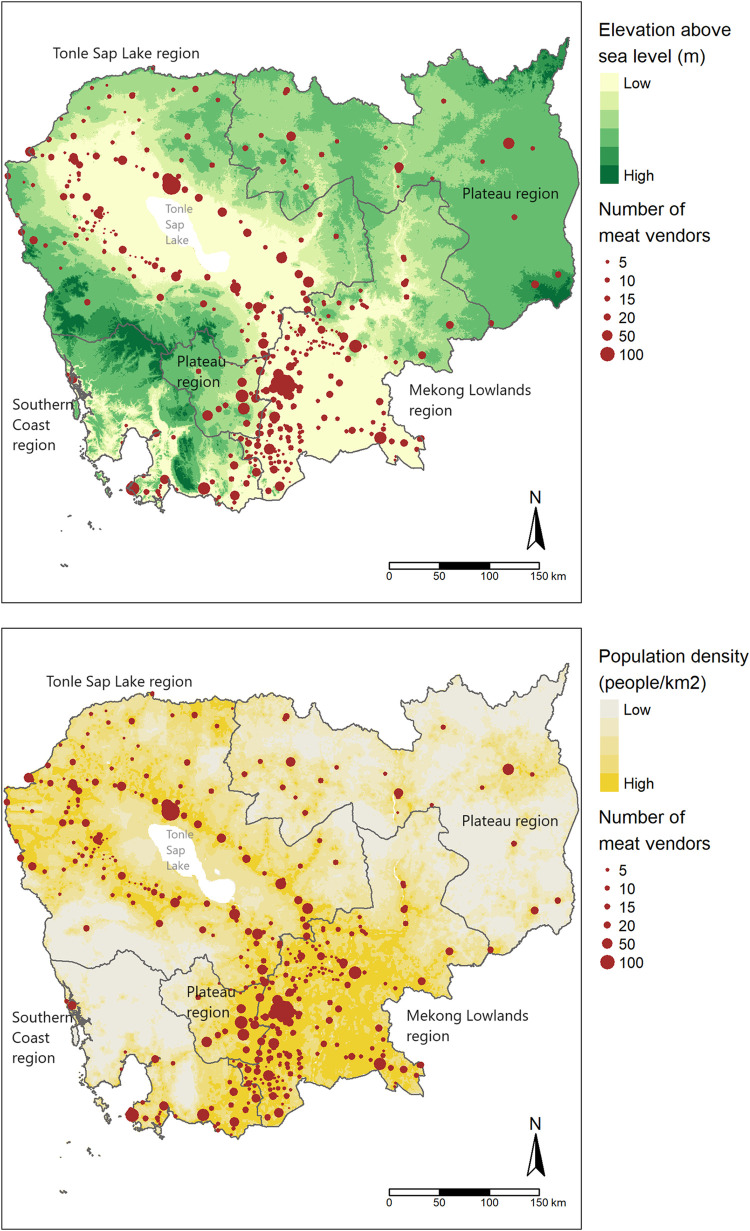
Food markets distribution in Cambodia with regional boundaries and (A) geographical characteristics or (B) population density. The base map of elevation and population density was reprinted with permission from WorldPop under a CC BY license. Cambodia has four geographical regions, including the Plateau region that comprises of two discontiguous polygons.

Fresh food markets were distributed unevenly in Cambodia (Fig 1). Markets were highly concentrated in the plain areas, typically in the center of the Mekong Lowlands or along the Tonle Sap Lake. These markets also had large number of meat vendors. In contrast, markets were sparse and small in size in areas with high elevation such as the Plateau region. On average, the density of food market was 9/1000 km^2^ in the Mekong Lowlands, 2.5/1000 km^2^ in the Tonle Sap Lake region and 0.5/1000 km^2^ in the Northeastern Plateau region (S1 Table). Nevertheless, food market distribution closely follows population distribution (Fig 1) with the market density varying slightly between 2.5 to 3.5 per 100,000 persons across the four regions.

**Fig 2:**
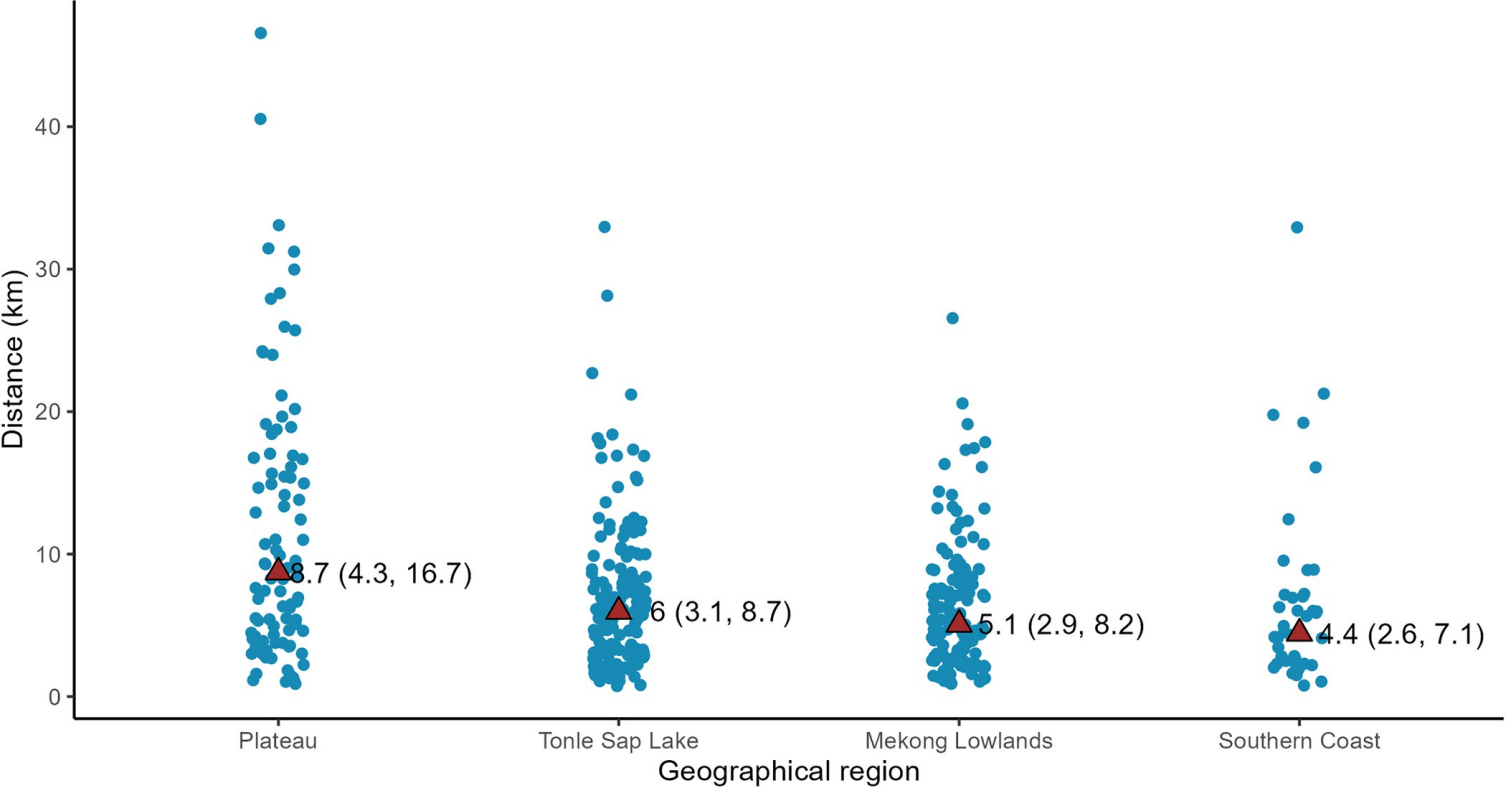
Straight-line distance from households to nearest markets. Households included all rural households enrolled into the Demographic and Health Survey, whether or not households had children under five years of age. Each dot represents a household and a random noise was added to points to increase the readability of the plot. Red triangle indicates regions-specific median of the distance to markets. Values are Median (IQR).

Average distance to nearest markets also differed across regions (Fig 2). On average, half of the rural households in the Mekong Lowlands, Tonle Sap Lake and Southern Coast regions lived within 4 km to the nearest food market (IQR ranges between 3 km to 8 km). The distance to food markets is a lot farther in the Northeastern Plateau with half of the households living within 9 km (IQR: 4 km to 17 km). Such regional estimates mask a significant within-region heterogeneity with many households across the four regions having to travel over 20 km to reach the nearest market.

### Associations between distance to markets and child nutrition indicators

The associations between distance to markets and child nutrition indicators were evaluated in our sample of children with dietary diversity data (n = 1537) or height-for-age z-score (n = 989) (Table 1). In the sample with dietary diversity data, the mean distance from households to the nearest market was 7.22 (SD: 5.98) km. The mean dietary diversity score was 3.20 (SD: 1.62; range: 1–7), which reflects the pattern in which only 60% of the children consuming fish and seafood, 45% consuming meat such as poultry and pork and about 30% or fewer consuming eggs, dairy or nuts (S1 Fig). In this sample, half of the children were male and mean child age was 14.38 (SD: 5.32) month. About 15% of the mothers had no education, half (50%) had incomplete or completed primary education and 30% had secondary education or higher. Three-quarters of the households owned less than 1 hectare of agricultural land while one-quarter did not own any livestock. In the sample with height data (n = 989), mean height-for-age z-score was -1.25 (SD: 1.31) and other covariates had similar covariate distribution as the sample of children with dietary diversity data, except for a higher proportion of households with mothers having at least some primary education (59.3% vs. 54.4%).

**Table 1:** Characteristics of the samples of children aged 6 to 23 months in rural Cambodia.

Sample characteristics	Sample with dietary diversity data (n = 1537) ^a^	Sample with height-for-age Z-score data (n = 989) ^a^
Distance to market (km), *mean (SD)*	7.22 (5.98)	7.31 (6.09)
Dietary diversity score, *mean (SD)*	3.20 (1.62)	
Height-for-age Z-score, *mean (SD)*		-1.25 (1.31)
Child age, *mean (SD)*	14.38 (5.32)	14.46 (5.21)
Child sex, n (%)		
Male	913 (50.3%)	67 (51.9%)
Female	903 (49.7%)	562 (48.1%)
Maternal age, n (%)		
15–24 y	657 (36.2%)	429 (36.7%)
25–30 y	627 (34.5%)	412 (35.3%)
>30 y	533 (29.3%)	327 (28.0%)
Maternal education, n (%)		
No education	266 (14.6%)	164 (14.0%)
Incomplete or completed primary education	988 (54.4%)	693 (59.3%)
Some secondary education or more	562 (31.0%)	312 (26.7%)
Household wealth, n (%)		
Low	546 (30.1%)	366 (31.4%)
Middle	582 (32.1%)	372 (31.8%)
High	688 (37.9%)	430 (36.8%)
Household size, n (%)		
< = 5 members	961 (52.9%)	635 (54.4%)
>5 members	855 (47.1%)	533 (45.6%)
Agricultural land ownership, n (%)		
< 1 hectare	1313 (72.3%)	861 (73.7%)
> = 1hectare	503 (27.7%)	307 (26.3%)
Tropical Livestock Unit ^b^, n (%)		
0 (Owning no animals)	464 (25.5%)	284 (24.3%)
0.01–0.1 (Mostly small livestock)	416 (22.9%)	287 (24.5%)
0.11–1.4 (Mixed of large and small livestocks)	468 (25.8%)	300 (25.7%)
> 1.4 (Many animals, mixed of large and small livestock)	469 (25.8%)	298 (25.5%)
Region, n (%)		
Mekong Lowlands	757 (41.7%)	387 (41.7%)
Tonle Sap Lake	643 (35.4%)	396 (33.9%)
Plateau	308 (17.0%)	210 (18.0%)
Southern Coast	107 (5.9%)	75 (6.4%)

^a^ Weighted sample size of the sample with diet and height data was 1816 and 1168, respectively.

^b^ Tropical Livestock Unit (TLU) is a livestock score that assigns different weighting factors to different livestock

Longer distance to the nearest market was associated with lower dietary diversity score in the univariate model, and the association was attenuated after adjusting for other predictors (β: -0.17; 95% CI: -0.29, -0.05) (Table 2). In the multivariate model, children who were older in age, lived in households in higher wealth tertiles, had mothers aged between 25 and 30 years or mothers with secondary education or above also had better dietary diversity compared to younger children, children from lower wealth tertiles, or children who had mothers aged younger than 25 years or mothers with no education. On the other hand, measures of land and livestock ownership were not associated with dietary diversity score.

**Table 2:** Associations of distance to nearest markets with children’s dietary diversity score and height-for-age z-score in rural Cambodia.

Predictors	Dietary diversity score (n = 1537)		Height-for-age z-score (n = 989)	
	Univariate model ^a^	Multivariate model ^b^	Univariate model ^a^	Multivariate model ^b^
(Log) distance to market	-0.30 (-0.42, -0.17) ***	-0.17 (-0.29, -0.05) **	-0.11 (-0.24, 0.01)	-0.01 (-0.13, 0.11)
Child age	0.13 (0.11, 0.14) ***	0.13 (0.11, 0.14) ***	-0.07 (-0.08, -0.05) ***	-0.06 (-0.08, -0.05) ***
Child sex, Female vs. Male	-0.05 (-0.22, 0.12)	-0.07 (-0.21, 0.07)	0.15 (-0.02, 0.32)	0.14 (-0.03, 0.31)
Maternal age, vs. 15-24y				
25–30 y	0.30 (0.08, 0.53) *	0.21 (0.01, 0.41) *	-0.03 (-0.23, 0.17)	-0.02 (-0.22, 0.18)
>30 y	0.17 (-0.08, 0.42)	0.09 (-0.12, 0.31)	-0.12 (-0.36, 0.12)	-0.08 (-0.31, 0.15)
Maternal education, vs. No education				
Incomplete or completed primary education	0.20 (-0.05, 0.44)	0.19 (-0.07, 0.44)	-0.02 (-0.31, 0.26)	-0.22 (-0.49, 0.04)
Some secondary or more	0.72 (0.39, 1.06) ***	0.53 (0.19, 0.86) **	0.07 (-0.24, 0.39)	-0.17 (-0.48, 0.14)
Household wealth, vs. Low				
Middle	0.38 (0.14, 0.63) **	0.40 (0.17, 0.62) ***	0.16 (-0.07, 0.40)	0.08 (-0.15, 0.30)
High	0.59 (0.30, 0.88) ***	0.43 (0.17, 0.69) **	0.51 (0.28, 0.74) ***	0.42 (0.16, 0.67) **
Household size, >5 members vs. < = 5 members	-0.16 (-0.36, 0.05)	-0.16 (-0.33, 0.02)	0.11 (-0.06, 0.28)	0.03 (-0.13, 0.19)
Agricultural land ownership, > = 1 hectare vs. < 1 hectare	0.19 (-0.05, 0.42)	0.18 (-0.02, 0.38)	-0.15 (-0.36, 0.06)	-0.18 (-0.39, 0.04)
Tropical Livestock Unit ^c^, vs. No livestock				
0.01–0.1 (Mostly poultry)	-0.08 (-0.41, 0.24)	-0.04 (-0.31, 0.23)	-0.31 (-0.57, -0.06) *	-0.20 (-0.46, 0.05)
0.11–1.4 (Mixed of large & small animals)	0.11 (-0.23, 0.45)	0.10 (-0.16, 0.35)	-0.24 (-0.50, 0.02)	-0.07 (-0.34, 0.19)
> 1.4 (Many mixed of large & small animals)	-0.05 (-0.36, 0.26)	-0.06 (-0.32, 0.20)	-0.01 (-0.29, 0.26)	0.11 (-0.18, 0.39)
Region, vs. Mekong Lowlands				
Tonle Sap Lake	0.17 (-0.09, 0.43)	0.17 (-0.04, 0.38)	-0.12 (-0.36, 0.11)	-0.01 (-0.24, 0.22)
Plateau	-0.03 (-0.36, 0.30)	0.09 (-0.21, 0.39)	-0.35 (-0.65, -0.05) *	-0.30 (-0.57, -0.02) *
Southern Coast	0.02 (-0.33, 0.37)	-0.02 (-0.32, 0.29)	0.32 (-0.05, 0.69)	0.24 (-0.12, 0.61)

Values are Mean different (95% Confidence interval), which were obtained from univariate or multivariate linear regression that accounts for Demographic and Health Survey’s complex survey design. P-value:

*P < 0.*05*

**P < 0.01

***P < 0.001

^a^ The univariate models evaluate each predictor at a time.

^b^ The multivariate model include all predictors showed in this table in the model.

^c^ Tropical Livestock Unit (TLU) is a livestock score that assigns different weighting factors to different livestock

We found no association between distance to nearest markets and height-for-age z-score before (β: -0.11; 95% CI: -0.24, 0.01) or after adjusting for other predictors (β: 0.00; 95% CI: -0.13, 0.11). Rather, being born to households in the highest wealth tertile was associated with higher z-score compared to children born to households in the lowest wealth tertile. In contrast, children who were older in age or lived in the Plateau region had lower z-score than younger children or children living in the Lowlands. In all multivariate models, VIFs were below 2, suggesting that multicollinearity was not present.

In the univariate models predicting individual food consumption (secondary outcomes), we found that only consumption of egg, dairy, meat, vitamin A fruits and other fruits and vegetables were associated with log-transformed distance to the nearest market. We then run the multivariate model only for these five individual food accounting for covariates indicated in the Table 2. These multivariate models showed that only the consumption of meat and other fruits and vegetables were predicted by distance to markets (S2 Table).

### Effect modifications of market access with child diet by household wealth

**Fig 3:**
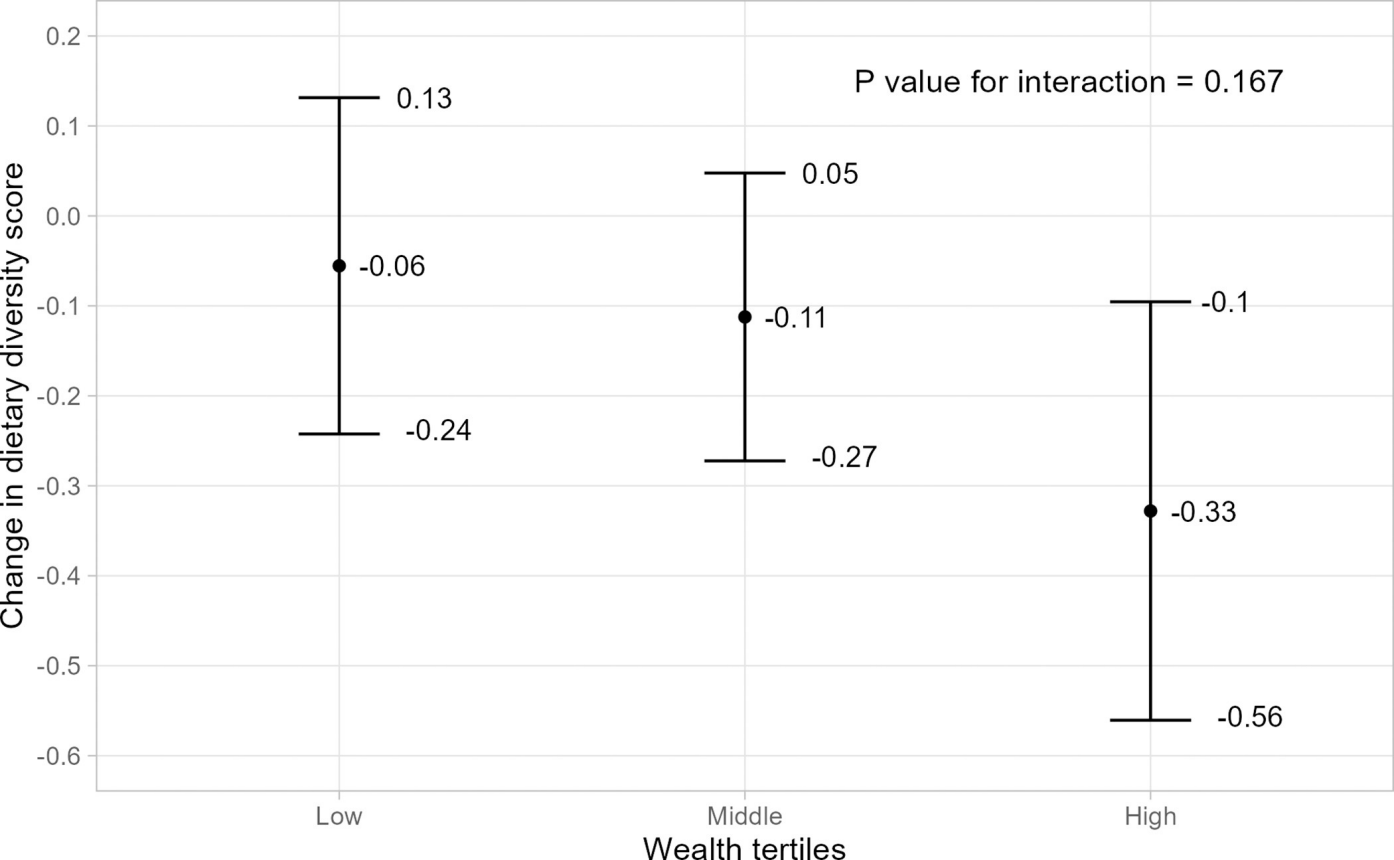
Associations between distance to market and dietary diversity score by household wealth tertiles. Notes: Error bars are 95% CIs of the marginal trends obtained from the post-hoc estimation of the multivariate linear regressions that included all covariates in Table 2 and an interaction term of distance to market and household wealth. The overall P-value was obtained from the Type 3 test.

Analyses evaluating effect modification were carried out only for dietary diversity score, the primary outcome for which we observed a main effect. Our results showed that the inverse association between distance to markets and dietary diversity score was found for the high wealth tertile (β: -0.33; 95% CI: -0.56, -0.10), but not the middle (β: -0.11; 95% CI: -0.27, 0.05) and the low tertile (β: -0.05; 95% CI: -0.24, 0.13) (Fig 3). Nevertheless, the overall interaction was non-significant (P-value = 0.165).

## Discussion

This present study leverages a national listing of food markets in Cambodia to assess the relationship between food market access and child nutrition indicators. Food markets in Cambodia were distributed unevenly with large markets concentrating in the populated areas in the low lands and small markets sparsely distributed in the remote, plateau areas. Distance to food markets was associated with better dietary diversity but not height-for-age z-score among children under two years of age in the rural Cambodia, and there was no evidence that household wealth modified the associations between distance to food market and child dietary diversity.

We show that children who lived closer to markets had better dietary diversity than children who lived farther. This finding is consistent with previous research, which showed better child’s dietary diversity with shorter distance to market [22], shorter time travelled to market [20], lower transportation cost to markets [18, 19] or better food variety sold in nearest markets [20]. However, the magnitude of these associations was small. For example, in Ethiopia, child dietary diversity score during the lean season differed by 0.73 between households living within 3 km and households living further than 3 km from the nearest market [22]. Another study in Ethiopia indicates that, after controlling for distance to markets, increasing the number of food groups sold in the market from three to six was associated with a small increase of 0.27 in the number of foods consumed by the children [20]. In our study, the magnitude of the association was also small, such that increasing the distance in natural log scale by a unit (i.e., increasing the distance by 2.7 times) was associated with a decrease of 0.17 in the dietary diversity score.

We initially hypothesized that household wealth modifies the association between market access and child diet, because low-income households may not be able to afford nutritious food even if nutritious food are available in the close neighborhood. However, this hypothesis is not supported by our findings of non-significant effect modification by wealth. One possible explanation is that, regardless of the wealth status, rural households in Cambodia acquire food from other sources and their dietary diversity is thus less sensitive to the cost of diet as we initially hypothesized. Although we lack household food acquisition data, we find that distance to market only predicted the consumption of meat (including pork, beef and poultry) and other fruits and vegetables. Other food groups, which were not related to distance to markets, were possibly acquired from other sources. For example, fish and seafood, a food group consumed by 60% of the children in our sample, is commonly wild caught from lakes or paddy rice fields for subsistence in rural Cambodia [46]. Vitamin A-rich vegetables such as spinach, bok choy, pumpkins and carrots might also be readily available in the backyards for home consumption [47]. In the future studies, a detailed household survey that captures sources of acquisition for different household food items is needed to understand how high-income and low-income households differ in their utilization of food markets.

We also found that child height-for-age z-score was not related to distance to nearest market. This finding aligns with the two prior studies that found null associations between distance to markets and child height in Ethiopia [18, 22]. It is possible that the effects of market access on dietary diversity is not large enough to lead to significant improvement in height. Furthermore, linear growth is a complex process influenced by intrauterine growth, breastfeeding practices and exposure to pathogens and environmental contaminants [48], which are not amenable through improving market access alone.

Our findings have implications for future nutrition policies and programs in Cambodia. The modest relationship between distance to market and child diet found in this study and prior literature suggests that investments on road and market infrastructure alone might not yield meaningful impact on child diet. In parallel to these investments, programs and policies should seek to strengthen support for non-market food sources such as home gardens, wild caught fisheries and mobile food traders. In particular, explicit dietary and nutrition outcomes should be integrated into the country’s agricultural and fisheries strategies through better nutrition mainstreaming [49]. More importantly, mobile food trader network has largely been absence from the current policy dialogues despite their potential role in within-rural food linkages [30]. Supporting these various sources of food acquisition also aligns with the current emphasis on food environment’s diversity and flexibility to help households better buffer potential socioeconomic and environmental shocks [50].

The strength of this study lies in its coverage of large geographical regions and socioeconomic groups in Cambodia, which was enabled by using the unique national census of meat-selling food markets and the novel approach to geocode food markets. Food markets geocoding remains an under-utilized tool in food access research but it is a promising approach because it saves the cost of conducting field work and market survey. In our study, we managed to geocode 485 out of 505 markets (96%), suggesting that this approach is promising and capable in characterizing the neighborhood food access. The study also has several limitations. First, it is not possible to ascertain the causality relationship between distance to markets and child dietary diversity due to the cross-sectional design of this analysis. Second, there was a four-year gap between the time the food market listing was compiled (2019) and the time when the household survey was carried out (2015). Nevertheless, food markets in Cambodia are structured according to administrative divisions, which were relatively stable over the course of five years. Furthermore, an earlier report estimated that there were 500 food markets in Cambodia in 2010 [30], which was close to the number of food markets listed in our input data (n = 503). Lastly, we lack information on which food, how much and at which cost households purchase from food markets versus other sources, which would help clarify the causal relationship between market access and child nutrition.

## Conclusions

In conclusion, our study shows that distance to food markets was weakly associated with child dietary diversity and it was not related to child height. These results suggest cautious optimism about the benefits of investment on road and market infrastructure and support other forms of food acquisition, such as subsistence fishing, subsistence gardening and food purchase from mobile food traders. These forms of food acquisition should be recognized and supported in the country’s policies on agriculture and rural development. Future research should clarify the role of food markets relative to other food sources through detailed food acquisition surveys.

## Supporting information

S1 Fig Percentage of children aged 6–23 months who consumed individual food groups. (DOCX)

S1 Table Market density by region and province in Cambodia. (DOCX)

S2 Table Estimates (95% CI) of the association between natural log-transformed distance to market and child consumption of individual food groups. (DOCX)
