# Supplementary figures and images for: Access to food markets, household wealth and child nutrition in rural Cambodia: Findings from nationally representative data
Source: PLoS One. 2023 Oct 18;18(10):e0292618. doi: 10.1371/journal.pone.0292618 (PMC10584123; doi:10.1371/journal.pone.0292618)

**Figure S1. Percentage of children aged 6- 23 months who consumed individual food**

**
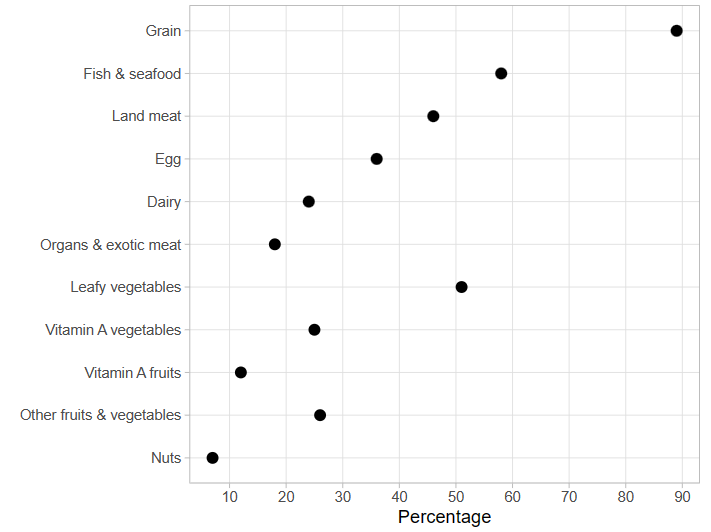
**

Supplement: S1 Fig — (DOCX) [file pone.0292618.s001.docx]
