# Supplementary material for: Access to food markets, household wealth and child nutrition in rural Cambodia: Findings from nationally representative data
Source: PLoS One. 2023 Oct 18;18(10):e0292618. doi: 10.1371/journal.pone.0292618 (PMC10584123; doi:10.1371/journal.pone.0292618)
Supplement: S1 Table — (DOCX) [file pone.0292618.s002.docx]

## Table S1. Market density by region and province in Cambodia

| **Region and provinces** | **Number of markets** | **Number of markets per 1000 km2** | **Number of markets per 100,000 persons** |
| --- | --- | --- | --- |
| **Mekong Lowlands region** | **226** | **9.0** | **3.0** |
| Takeo | 58 | 16.5 | 6.5 |
| Kampong Cham | 52 | 11.5 | 6.0 |
| Kandal | 43 | 12.0 | 3.5 |
| Phnom Penh | 37 | 99.0 | 1.5 |
| Svay Rieng | 18 | 6.5 | 3.5 |
| Tboung Khmum | 9 | 2.0 | 1.0 |
| Prey Veng | 8 | 1.5 | 1.0 |
| **Tonle Sap Lake region** | **170** | **2.5** | **3.5** |
| Battambang | 56 | 4.5 | 5.5 |
| Banteay Meanchey | 28 | 4.5 | 3.5 |
| Kampong Chhnang | 23 | 4.5 | 4.5 |
| Siem Reap | 20 | 1.5 | 2.0 |
| Oddar Meanchey | 15 | 3.0 | 5.5 |
| Kampong Thom | 14 | 1.0 | 2.0 |
| Pursat | 11 | 1.0 | 2.5 |
| Pailin | 3 | 3.0 | 4.0 |
| **Southern Coast region** | **29** | **2.0** | **3.5** |
| Preah Sihanouk | 15 | 15.5 | 5.0 |
| Kampot | 15 | 3.0 | 2.5 |
| Koh Kong | 6 | 0.5 | 5.0 |
| Kep | 3 | 20.0 | 7.0 |
| **Plateau region** | **50** | **0.5** | **2.5** |
| Kampong Speu | 21 | 3.0 | 2.5 |
| Preah Vihear | 10 | 0.5 | 4.0 |
| Stung Treng | 7 | 0.5 | 4.5 |
| Kratie | 6 | 0.5 | 1.5 |
| Mondul Kiri | 4 | 0.5 | 4.5 |
| Ratanak Kiri | 3 | 0.5 | 1.5 |
