# Supplementary material for: Access to food markets, household wealth and child nutrition in rural Cambodia: Findings from nationally representative data
Source: PLoS One. 2023 Oct 18;18(10):e0292618. doi: 10.1371/journal.pone.0292618 (PMC10584123; doi:10.1371/journal.pone.0292618)
Supplement: S2 Table — (DOCX) [file pone.0292618.s003.docx]

**Table S2: Estimates (95% CI) of the association between natural log-transformed distance to market and child consumption of 11 food groups**

|  | Univariate model | | Multivariate models | |
| --- | --- | --- | --- | --- |
| Food groups | β (95%CI) | P-value | β (95%CI) | P-value |
| Land meat (pork, poultry, beef, etc.) | -0.09 (-0.13; -0.06) | 0.000 | -0.05 (-0.09; -0.01) | 0.009 |
| Egg | -0.06 (-0.09; -0.02) | 0.003 | -0.03 (-0.06; 0.01) | 0.131 |
| Dairy | -0.07 (-0.10; -0.03) | 0.000 | -0.01 (-0.04; 0.02) | 0.597 |
| Other fruits & vegetables | -0.07 (-0.11; -0.04) | 0.000 | -0.05 (-0.08; -0.01) | 0.009 |
| Vitamin A fruits | -0.02 (-0.08; -0.01) | 0.015 | -0.02 (-0.06, 0.01) | 0.173 |
| Fish & seafood | 0.02 (-0.02; 0.05) | 0.431 |  |  |
| Organ & exotic meat | -0.01 (-0.04; 0.02) | 0.405 |  |  |
| Vitamin A vegetables | -0.02 (-0.05; 0.02) | 0.295 |  |  |
| Leafy vegetables | -0.02 (-0.06; 0.01) | 0.168 |  |  |
| Nuts | -0.02 (-0.04; 0.00) | 0.059 |  |  |
| Grain | -0.01 (-0.04; 0.01) | 0.186 |  |  |
